# Leptin-Induced Angiogenesis of EA.Hy926 Endothelial Cells *via* the Akt and Wnt Signaling Pathways *In Vitro* and *In Vivo*

**DOI:** 10.3389/fphar.2019.01275

**Published:** 2019-10-31

**Authors:** Fei Yu, Runqing Fu, Lu Liu, Xiaoting Wang, Tingting Wu, Wei Shen, Zhipeng Gui, Xiumei Mo, Bing Fang, Lunguo Xia

**Affiliations:** ^1^Department of Orthodontics, Ninth People’s Hospital Affiliated to Shanghai Jiao Tong University School of Medicine, Shanghai Key Laboratory of Stomatology, Shanghai, China; ^2^College of Chemistry, Chemical Engineering and Biotechnology, Donghua University, Shanghai, China; ^3^Department of Oral Surgery, Ninth People’s Hospital Affiliated to Shanghai Jiao Tong University School of Medicine, Shanghai Key Laboratory of Stomatology, Shanghai, China

**Keywords:** angiogenesis, leptin, poly(L-lactide-co-caprolactone), electrospinning, tissue engineering

## Abstract

Angiogenesis involves the activation of endothelial cells followed by capillary formation. Leptin, the protein product of the ob gene, can induce the angiogenic potential of endothelial cells. However, the underlying cellular mechanism still remains to be elicited. We firstly evaluated the *in vitro* effects of leptin on proliferation and angiogenic differentiation of endothelial cell line EA.hy926. Leptin was found to potently induced cell proliferation, expression of angiogenic gene, migration and tube formation. Then we investigated the roles of the Akt and Wnt signaling pathways in the aforementioned processes. It showed that Akt and Wnt signaling pathways could be activated by leptin, while inhibition of the Akt and Wnt signaling pathways by siRNAs effectively blocked the leptin-induced angiogenesis. Finally, we used electrospinning to fabricated leptin-immobilized linear poly(L-lactide-co-caprolactone) (PLCL)-leptin. The *in vivo* vessel formation of PLCL-leptin was evaluated using subcutaneous implants in Sprague-Dawley rats. The histological and immunofluorescence revealed that cell infiltration with PLCL-leptin was much more significant than that with the control PLCL group. More importantly, the number of laminin^+^ vessels and CD31^+^ cells in PLCL-leptin grafts was significantly higher than in control grafts. The study demonstrated that it is *via* Akt and Wnt signaling pathways that leptin promotes the proliferation and angiogenic differentiation of endothelial cells and the capacity of endogenous tissue regeneration makes the novel leptin-conjugated PLCL promising materials for grafts.

## Introduction

Cardiovascular diseases produce serious health problems to the humans worldwide (Roth et al., 2015). Artificial vascular grafts are with success utilize in clinical applications for large-diameter blood vessels, but fail attribute to the poor patency used as small- to medium-sized grafts (SMGs) (Zilla et al., 2007). Angiogenesis is a physiological process that includes the activation of endothelial cells followed by the development of new capillary vessels (Maragoudakis, 2000). It is a fundamental biological process indispensable for tissue regeneration. A set of chemokines, such as stromal cell-deceived factor-1 alpha (SDF-1α) and granulocyte colony stimulating factor (G-CSF) have been revealed to recruit endogenous mesenchymal stem cells, endothelial progenitor cells, and smooth muscle progenitor cells (Lee et al., 2013; Krieger et al., 2016), which make them the target of However, they often fail to conjugated with scaffolds, and they are easily cleaved (Shafiq et al., 2015). Besides, some growth factors, such as vascular endothelial growth factor (VEGF), epidermal growth factor (EGF), Hepatocyte growth factor (HGF) have been studied in angiogenesis (Ding et al., 2003; Hoeben et al., 2004; van Cruijsen et al., 2005). However, because of cost, biological half-life, uncertain side effect limited their clinical application (Epstein et al., 2001; Lee et al., 2003; Taraboletti and Giavazzi, 2004).

Leptin, a 146-amino acid polypeptide secreted by adipocytes, is the protein product of the ob gene (Green et al., 1995). Leptin has a crucial status in the regulation of food intake and body weight. For vessel regeneration, leptin also demonstrates its potential ability (Sierra-Honigmann, 1998) and tube-like structure-inducing ability *in vitro* (Wolk et al., 2005). Endothelial cells expresses leptin receptors, and forms tube structures when stimulated by leptin (Bouloumie et al., 1998; Sierra-Honigmann, 1998). *In vivo* experiments confirm that leptin influences angiogenesis in chick chorioallantoic membranes (Bouloumie et al., 1998) and the disc angiogenesis system (Anagnostoulis et al., 2008). However, the underlying mechanisms should be much clarified. Leptin triggers STAT3, PI3K, extracellular signal-regulated kinase (ERK) and protein kinase A (PKA) signaling pathways after the activation of endothelial leptin receptor (Ob-R)(Rahmouni and Haynes, 2005). Some studies suggest that blocking the STAT3 pathway downregulates vascular endothelial growth factor (VEGF) mRNA expression (Suganami et al., 2004), while others demonstrated that leptin activates the PI3K-Akt pathway during endothelial migration (Goetze et al., 2002). With fragment studies, the fulfilled and thorough research towards mechanism of leptin-promoted angiogenesis is warranted. Akt promotes cell viability by phosphorylating downstream targets, such as glycogen synthase kinase-3β (GSK-3β). GSK-3β is inactivated when phosphorylated at the Ser9 residue, which in turn promotes the stabilized state of β-catenin. Furthermore, β-catenin is a crucial component of the canonical Wnt pathway. The translocation of β-catenin to the nucleus can result in a cycle of gene transcription and endothelial proliferation. Also, β-catenin is considered critical for vascularization in the central nervous system (Stenman et al., 2008). In recent years, the Wnt signaling pathway has drawn much attention as a regulator of vascular homeostasis and a determinant in vascular diseases (Shi et al., 2017). Endothelial cells express various Wnt ligands and their frizzled receptors, which simulate endothelial proliferation (Carmeliet and Jain, 2011). The β-catenin/Tcf-Lef reporter activity was reported to promote VEGF expression in tumor cells (Fernández et al., 2014). In addition, VEGF and the Wnt signaling pathway are also closely related (Maes et al., 2010). Here, we hypothesize that the key mechanism for the crucial angiogenesis function of leptin is the activation of the Akt and Wnt/β-catenin signaling pathways. Furthermore, whether leptin induces the angiogenesis process by activating crosstalk between the Akt and Wnt/β-catenin signaling pathways is undefined, and the present study also aims to discuss the relationship between leptin and the Akt and Wnt/β-catenin signaling pathways.

Considering the pharmacologic intervention of leptin could have uncertain side-effect on other tissue (Guo et al., 2012; Delort et al., 2015), we purposed to use leptin locally in tissue engineering. The multifunctional cellular effects of leptin, combined with poly(L-lactide-co-caprolactone) (PLCL) materials, could be very useful for the remodeling of vascular grafts through host cell recruitment, cell proliferation and cell differentiation. Sustainable release of drugs is difficult for vascular grafts, because fast release induces incomplete tissue regeneration of the surrounding tissue. The PLCL grafts, which were fabricated by coaxial electrospinning in the present study, can encapsulate drugs into the fibers, extending drug release time (Huang et al., 2009; Huang et al., 2013; Yin et al., 2017). Therefore, leptin could be stably released *in vivo* over an extended period of time. Leptin loaded onto PLCL with coaxial electrospinning could be released with a sustainable rate *in vivo*, consequently promoting angiogenesis.

In the present study, we methodically demonstrated the effects of leptin on the proliferation and angiogenesis processes of endothelial cells with a special light cast on the cellular mechanism underlying leptin-induced angiogenesis and the crosstalk between the Akt and Wnt signaling pathways *in vitro*. Also, we intended to design electrospun PLCL scaffolds that are conjugated with leptin for as artificial vascular grafts and characterize their revascularization ability *in vivo*.

## Materials and Methods

### Cell Culture

EA.hy926 endothelial cells (Shanghai Institutes for Biological Sciences, China Academy of Sciences, Shanghai, China) were cultured in DMEM (Gibco, USA) supplemented with 10% fetal bovine serum (Gibco, USA), 100 U/ml penicillin (HyClone, Thermo Scientific, USA) and 100 μg/ml streptomycin (HyClone, Thermo Scientific, USA). The EA.hy926 endothelial cells were cultured in humidified air with 5% CO_2_ at 37°C.

### Cell Proliferation Assay

The viability of cells was assessed using the Cell Counting Kit-8 (CCK-8) assay (Dojindo Laboratories, Kumamoto, Japan). EA.hy926 endothelial cells were seeded into a 96-well plate at the density of 3 × 104 cells per well with various concentrations of leptin (0, 0.1, 1, 5, 10, or 50 ng/ml). Premixed CCK-8 (10 μl) and medium were added to the 96-well plates, and cells were then incubated for 1 h at 37°C. The values of A450 were obtained with the 3550 automatic detector from Beckman (Brea, CA).

### Quantitative Real-Time Polymerase Chain Reaction

Total RNA was extracted from cultured cells using Trizol (Invitrogen, New York, USA), and then 1 μg of purified total RNA was incubated with the kit, PrimeScriptTM Master Mix (Takara, Kyoto, Japan), in a final volume of 20 μl to generate complementary DNA. The SYBR Green kit (Takara, Kyoto, Japan) was applied to conduct a quantitative real-time polymerase chain reaction (PCR) assay consisting of a denaturation step at 95°C for 30 s, followed by 5 s of annealing at 95°C and 40 s of elongation at 60°C for 40 cycles on an ABI Q6 real-time quantitative PCR instrument (ABI, Foster, USA). The primers for amplification were as shown in **Table 1**. The data were subjected to the comparative cycle threshold method (ΔΔCt) and normalized to the housekeeping gene, β-actin.

**Table 1:** Primers used in quantitative real-time PCR.

Primers	Forward Sequences (5’ to 3’)	Reverse Sequences (5’ to 3’)
β-*actin*	GGACTTCGAGCAAGAGATGG	AGCACTGTGTTGGCGTACAG
*VEGF*	CTACCTCCACCATGCCAAGT	CACACAGGATGGCTTGAAGA
*CD31*	TGCAGTGGTTATCATCGGAGTG	CGTTGTTGGAGTTCAGAAGTG
*CD144*	TCACCTGGTCGCCAATCC	AGGCCACATCTTGGGTTCCT
*KDR*	GTGATCGGAAATGACACTGGAG	CATGTTGGTCACTAACAGAAGCA
*PDGF*	ACGTGAGGAAGAAGCCAAAA	TCTGGTTGGCTGCTTTAGGT
*Wnt1*	TGGTGCTGCCACGTCAGCTG	TCCGGGGCAGGTCCATCAGG
*Wnt3a*	AGATGGTGGTGGAGAAGCAC	GTAGCAGCACCAGTGGAACA
*Wnt5a*	CATGAACCTGCACAACAACG	GCCAGCATGTCTTCAGGCTA

### Scratch Healing Assay

Cells were seeded into 12-well culture plates at 3 × 105 cells per well. After 24 h growth, the monolayer was scratched with a sterile 20 μl pipette tip across the center of the well in a straight line. After scratching, the wells were washed with PBS twice to remove detached cells. Then, basic medium without FBS and with various concentrations of leptin were added prior to incubation. After 24 h, migratory cells were photographed by an inverted microscope, and five fields were counted for each well. The migrating area was measured by ImageJ software (NIH), and migrating area (mm^2^) = A_0_-A_24_, where A_0_ represents the initial wound area (t = 0 h), A_24_ represents the wound area at 24 h.

### Tube Formation Assay

The Matrigel tube formation assay was also performed to assess *in vitro* angiogenesis (Arutyunyan et al., 2016). Growth factor-reduced Matrigel (BD) was placed in 96-well culture plates (50 μl per well) and allowed to rest at 37°C for 40 min. Then, 3 × 104 EA.hy926 endothelial cells were added to each well and incubated in basic medium with various concentrations of leptin for 9 h. Endothelial cells forming capillary-like tubes were photographed with an inverted microscope (Nikon Eclipse 80i; Nikon, Tokyo, Japan) and an imaging software NIS-Elements D 4.50 (Nikon Instruments, Tokyo, Japan). Five fields were counted for each well. The length of the tube was measured by ImageJ software (NIH).

### Cell Apoptosis Assay

A commercial kit was used for cell apoptosis assay (BD, Franklin Lakes, NJ, USA). After treating with different concentrations of leptin for 24 h, the cells and the cellular supernatant were collected and suspended in 500 μl of binding buffer. 5 μl of Annexin-V-fuorescein isothiocyanate and 5 μl of propidium iodide were added into buffer, incubating for 15 min in dark. Flow cytometry analysis was performed to detect apoptotic cells.

### Western Blot Analysis

EA.hy926 endothelial cells were seeded into 6-well culture plates with 6× 105 cells per well. After 24 h culture time, the cells were treated with 5 ng/ml leptin for several time periods. After treatment, cells were lysed with RIPA buffer supplemented with different inhibitors (1 mM NaVO4, 10 mM NaF, or 1% phosphatase inhibitor cocktail mixed with 1% protease inhibitor cocktail). In addition, nuclear proteins were extracted with nuclear fractionation protocol. Briefly, the cells were lysed in the following buffer: 10 mM HEPES, 1.5 mM MgCl_2_, 10 mM KCl, 0.5 mM DTT, 0.05% NP-40. After 10 min on ice, samples were centrifuged for 10 min at 3000 rpm. Pellets were resuspended in 400 μl of buffer containing 5 mM HEPES (PH 7.9), 1.5 mM MgCl_2_, 0.2 mM EDTA, 0.5 mM DTT, 26% glycerol, 4.6 M NaCl. The suspension was mixed vigorously and incubated on ice for 30 min, then was centrifuged at 24,000 g for 20 min at 4°C. The supernatant was used as a nuclear fraction. Proteins samples were mixed with SDS loading buffer, heated, and then separated by 10% or 12% SDS-polyacrylamide gel electrophoresis (SDS-PAGE) before transfer to Polyvinylidene fluoride (PVDF) membranes. The membranes were blocked with 5% BSA in TBST (5% w/v BSA, 1× TBS, 0.1% Tween-20), and probed with primary monoclonal antibodies against VEGF, platelet-derived growth factor (PDGF) (Abcam, Cambridge, MA, USA), kinase insert domain receptor (KDR), CD31, CD144, Akt, p-Akt, GSK-3β, p-GSK-3β, β-catenin, Glyceraldehyde 3-phosphate dehydrogenase (GAPDH) (CST, Boston, USA) and Lamin-B1(Proteintech, Rosemont, USA), followed by exposure to a secondary anti-rabbit or anti-mouse IgG antibody (CST, Boston, USA). Protein bands were analyzed using ImageJ software (NIH). The average gray values of bands were calculated automatically. When the GAPDH and Lamin-B1 bands had an even gray value, the gray values of p-AKT, p-GSK-3β, nuclear β-catenin were normalized to those of Protein Kinase B (AKT), GSK-3β, and Lamin-B1, respectively.

### Transfection of siRNA and Antagonist Treatment

Akt and β-catenin small interfering RNAs (siRNAs) were designed and synthesized by Shanghai Genepharma (Shanghai, China). Cells were seeded into six-well culture plates at 5 × 105 cells per well before transfected with siRNAs (Akt siRNA, 50 nM; β-catenin siRNA, 50nM) using Lipofectamine2000 (Invitrogen, Carlsbad, CA, USA). About 48 h later, the knockdown efficiency was analyzed by quantitative real-time PCR or western blot. Also, samples treated with siRNAs and leptin were collected and investigated with quantitative real-time PCR or western blot. The tube formation and scratch healing assays were also evaluated. Further, to demonstrate the influence of antagonists on leptin-induced signaling pathways, Wnt pathway inhibitor Dickkopf-1 (Dkk-1, Sigma Aldrich, Darmstadt, Germany) were added to endothelial cells to block the Wnt signaling pathway. After treatment with dkk-1, 5 ng/ml leptin was added to the cells.

### *In Vivo* Scaffold Preparation

Poly(L-lactide-co-caprolactone) (PLCL) (Jinan Daigang Biomaterial Co., Ltd, Jinan, China), leptin, 1, 1, 1, 3, 3,3-hexafluoro-2-propanol (HFIP) (Shanghai Darui Fine Chemical Co., Ltd. Shanghai, China), and absolute ethyl alcohol (Changshu Hongsheng Fine Chemical Co., Ltd) were used. The nanofibrous tube scaffolds were produced using electrospinning technology. Tubes were designed to have an internal diameter of 3 mm and a thickness of 200 μm. The detailed electrospinning process has been previously described (Huang et al., 2009; Zhu et al., 2016). Briefly, PLCL was dissolved in HFIP to form an 11% solution as the sheath layer solution, which was left overnight. Then, leptin (1000 ng/ml) was diluted in absolute ethyl alcohol to form a 5 ng/ml solution as the core layer solution. The electrospinning setup consisted of a custom-made voltage power supply with an adjustable voltage output, a stainless steel coaxial needle, a syringe pump and a rotating collector. The applied voltage and the distance between the tip of the needle and the collector were maintained at 14 kV and 20 cm, respectively. The sheath and core solution flow rates were set as 1.0 ml/h and 0.2 ml/h, respectively. All the electrospinning processes were carried out at approximately 25°C and 50% relative humidity. The tubes of electrospun nanofibers were carefully stripped from the shaft and dried under a vacuum for at least 24 h to remove residual solvent before further use.

### Morphological Analysis of Scaffolds

The morphology of scaffolds was characterized by field emission scanning electron microscope (VEGA 3 XMU [LaB6] [Scanning electron microscopy (SEM)] & XFlash 6130 [EDS], Curtiss-Wright Surface Technologies, Paramus, NJ). All samples were sputter coated with gold prior to analysis. The diameter of the fibers was calculated by ImageJ software (NIH) and presented as the mean ± SD of 50 fibers.

### Implantation of Scaffolds

Sprague-Dawley rats (age = 7 weeks, weight = 200- 250 g, n = 6) were divided randomly into two groups to receive PLCL or PLCL-leptin vascular grafts and were monitored for 28 d. Six samples were implanted per group. The rats were anesthetized by intraperitoneal injection of pentobarbital (Nembutal 3.5 mg/100 g). Then, a longitudinal incision was made in the dorsal skin of the rats, and a muscle pocket was created by blunt dissection. Scaffolds were implanted in the space between the skin and deep muscle. Two samples were implanted per animal on the right and left side of the incision. After 28 d, the rats were sacrificed, and the vascular scaffolds in surrounding tissues were explanted. The grafts were fixed with 4% phosphate-buffered paraformaldehyde solution for 24 h and then embedded in paraffin. Paraffin blocks were sectioned into slices (thickness = 4 μm) for histological and immunofluorescence staining. The use of animals was approved by the ethics committee of Ninth People’s Hospital affiliated with the Shanghai Jiao Tong University School of Medicine.

### Histological Analysis of the Explanted Grafts

The cell infiltration and tissue regeneration abilities of the grafts were observed by histological analysis. Hematoxylin and eosin staining and Masson’s trichrome (MT) staining were conducted according to standard protocols. In MT staining, connective tissues were stained blue, nuclei dark red/purple, and the cytoplasm red/pink. The stained sections were observed with a light microscope (Nikon Eclipse 80i; Nikon, Tokyo, Japan). Boundaries were represented by dash lines.

### Immunofluorescence Analysis

After the antigen retrieval step using 1 mmol/L EDTA (pH 8.0), 4-μm-thick paraffin sections were incubated with the primary antibody for laminin and CD31 staining. Rabbit anti-laminin antibody (1:30 dilution; Sigma-Aldrich, St. Louis, MO, USA) and mouse anti-CD31 antibody (1:100 dilution; CST, Boston, USA) were diluted in blocking serum at 4°C overnight, washed with PBS three times, and then treated with Alexa 488-conjugated goat anti-rabbit antibody or Alexa 647-conjugated goad anti-mouse antibody (1:500 dilution; Abcam, Cambridge, MA, USA). Then, all sections were followed by DAPI (Invitrogen, Carlsbad, CA, USA) for nuclear staining. All immunofluorescence images were captured by a fluorescence microscope (Nikon Eclipse 80i; Nikon, Tokyo, Japan) and NIS-Elements D 4.50 (Nikon Instruments, Tokyo, Japan). The numbers of positive cells were counted in three randomly selected images from grafts were averaged and are presented as means ± SD. The blood vessels quantification was analyzed with Image J software practicing the “analyze particle”.

### Statistical Analysis

SPSS v. 18.0 statistical software (SPSS Inc., Chicago, IL, USA), run on the Apple macOS Sierra operating system, was used for to data processing and analysis. All data were expressed as the means ± SD. Statistical analysis was performed by analysis of variance, Student’s t-test. Values of p < 0.05 were considered statistically significant.

## Results

### Leptin Promoted Proliferation and Expression of Angiogenic Genes of Endothelial Cells

**Figure 1:**
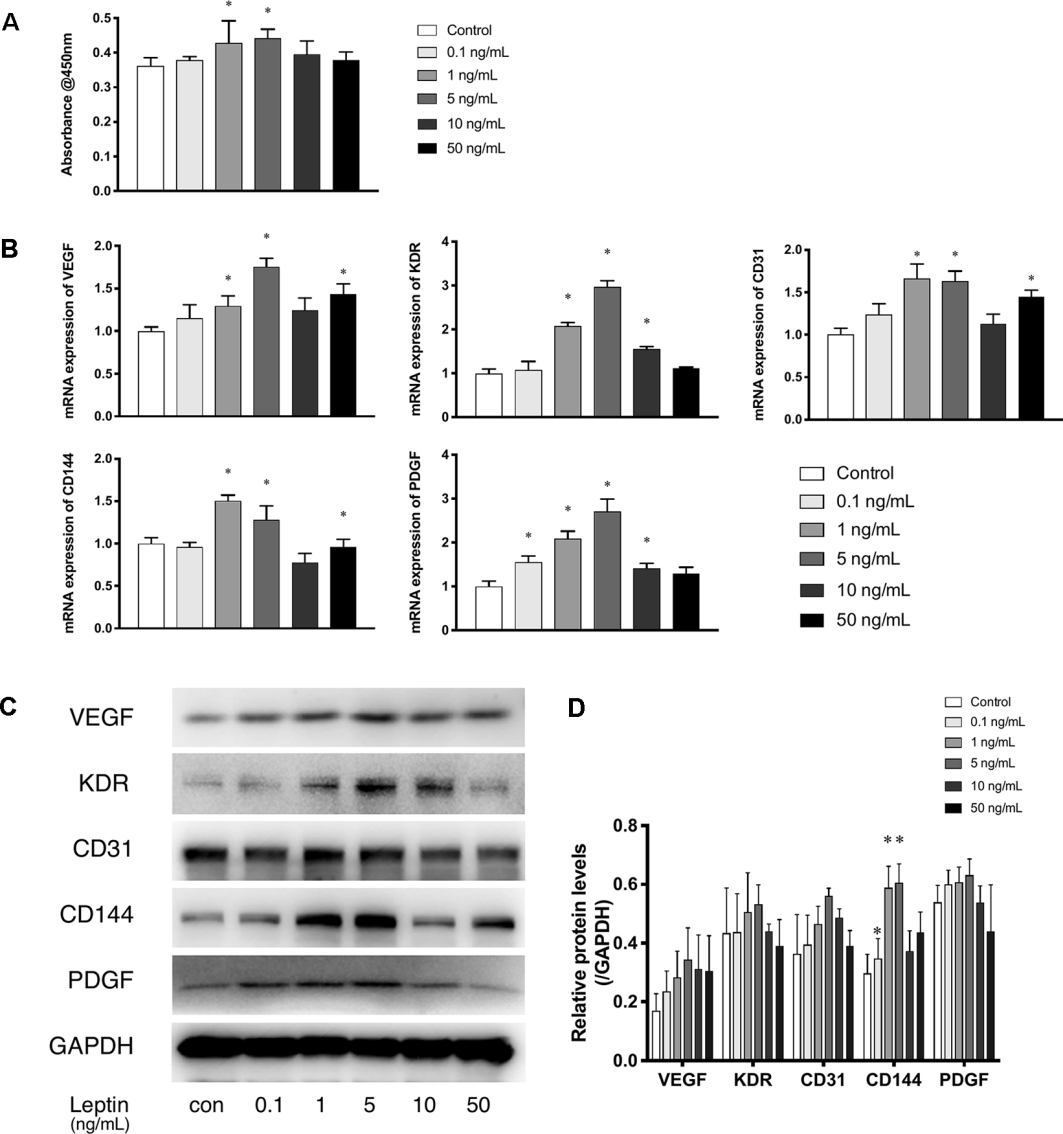
Leptin enhanced proliferation and angiogenesis gene expression of EA.hy926 endothelial cells. **(A)** Evaluation of the leptin-induced proliferation of EA.hy926 endothelial cells at 24h. The mRNA and protein expression of VEGF, KDR, CD31, CD144, PDGF was investigated by quantitative real-time PCR, which Data were normalized to β-actin control. **(B)** and Western blot assay **(B)**. **(B)** Relative protein levels of VEGF, KDR, CD31, CD144, PDGF, normalized with GAPDH in (Panel **C**). **(D)** Relative protein levels in (Panel C) of VEGF, KDR, CD31, CD144, PDGF, normalized with GAPDH respectively. *indicated significant differences between leptin-treated groups vs. control group. P < 0.05. The experiments were conducted in triplicate.

The CCK-8 assay was used as a visible assay to measure the proliferation of endothelial cells. The absorbance at 450 nm indicates the number of living cells. With increasing concentrations of leptin, the proliferation of endothelial cells exhibited a bell curve pattern. As shown in (**Figure 1A**), leptin promoted the proliferation of viable cells and significantly enhanced the growth of endothelial cells in a dose-dependent manner with a peak at the concentrations of 1 ng/ml and 5 ng/ml. These results indicated that cells proliferated in a dose-dependent manner. However, endothelial cells exposed to a high concentration of leptin (i.e., 10 ng/ml or 50 ng/ml) could had higher apoptosis rate than other concentrations (**Figure S1**). The expression patterns of related angiogenesis genes in endothelial cells cultured in different concentrations of leptin were analyzed using quantitative real-time PCR and western blot. Leptin increased the expression of VEGF, CD31, and CD144 genes at 1 ng/ml, 5 ng/ml and 50 ng/ml, compared with the control group (**Figures 1B–D**), that of kinase insert domain receptor (KDR) and platelet-derived growth factor (PDGF) genes at the concentrations of 1 ng/ml, 5 ng/ml, and 10 ng/ml, compared with control group. Over all, the increases were most remarkable in the 1 ng/ml and 5 ng/ml group.

### Leptin Enhanced Migration and Tube Formation of Endothelial Cells

**Figure 2:**
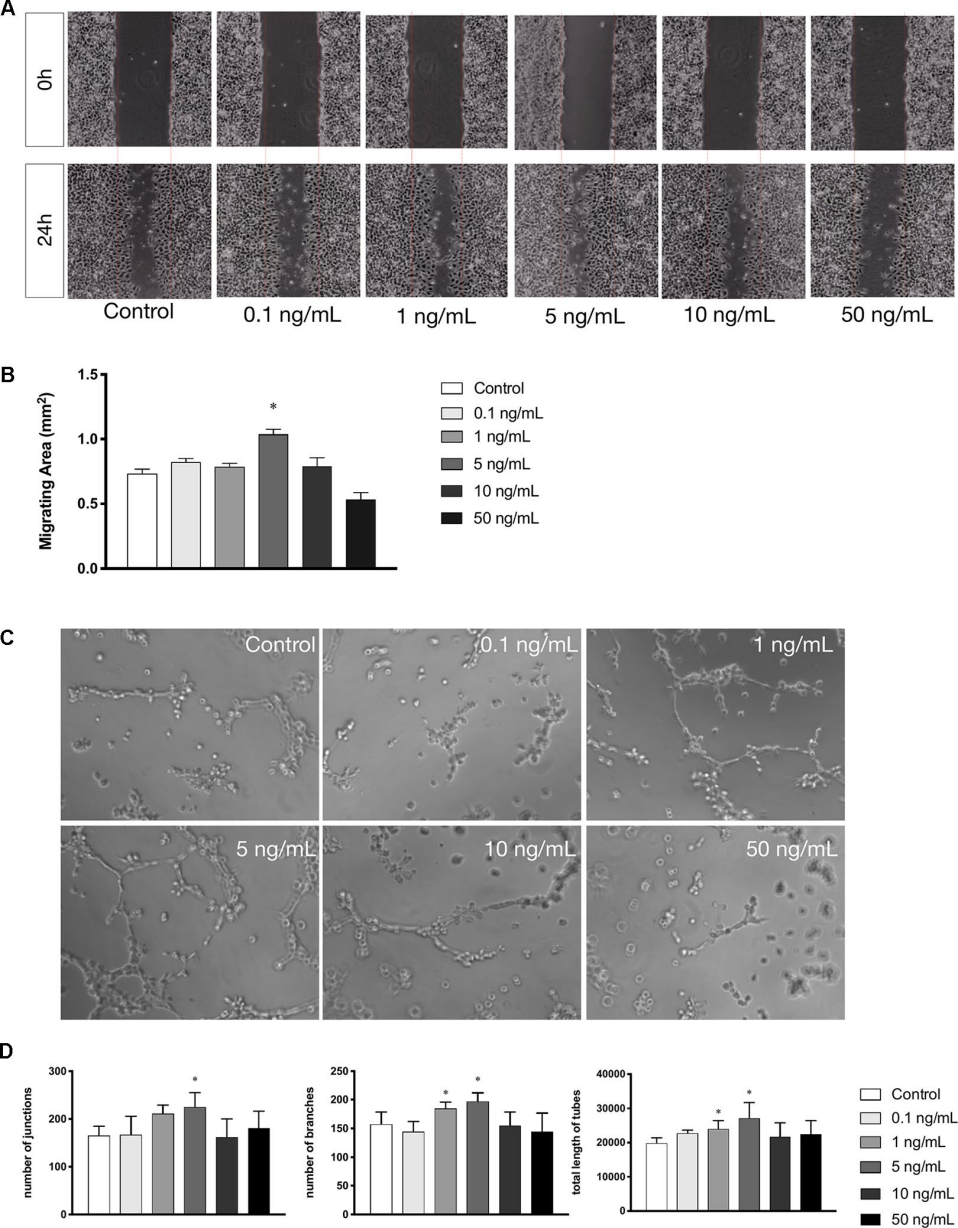
Leptin enhanced migration and tube formation of EA.hy926 endothelial cells. **(A)** Cells were treated with different concentrations of leptin and were performed wound healing assay (original magnification, 40×). The red lines indicated wound edge at 0h. **(B)** Migrating area (mm^2^) was calculated after 24h. **(C)** Cells were treated with different concentrations of leptin on BD Matrigel for 9h and capillary-like structures were examined (original magnification, 200×). **(D)** Number of junctions, number of branches and total length of tubes were valued. *indicated significant differences between leptin-treated groups vs. control group. P < 0.05. The experiments were conducted in triplicate.

Cell migration is another essential feature of vascular endothelial cells during angiogenesis. We investigated the effects of leptin on cell migration using the scratch healing assay. The migrating area was significantly overlapped by cell migration in non-serum conditioned medium with low concentrations of leptin. The percentage of migrating area significantly decreased with the addition of 5 ng/ml leptin. This result demonstrated that cell migration was magnified by 5 ng/ml leptin. Furthermore, the endothelial cells in a high concentration of leptin (i.e., 50 ng/ ml) could lose migration abilities (**Figures 2A, B**). Moreover, according to the tube formation assay, leptin played a key role in angiogenesis *in vitro*. The Matrigel-coated tube formation assay (**Figure 2C**) showed that EA.hy926 endothelial cells began to form polygon structures with 1 ng/ml and 5 ng/ml leptin. In addition, cells extended more branches and formed more tubes in the low leptin concentration groups, especially 5 ng/ml, based on the number of junctions, the number of branches and the total length of tubes statistically analyzed (**Figure 2D**).

### Effects of Leptin Were Observed Through the Akt and Wnt Pathways

**Figure 3:**
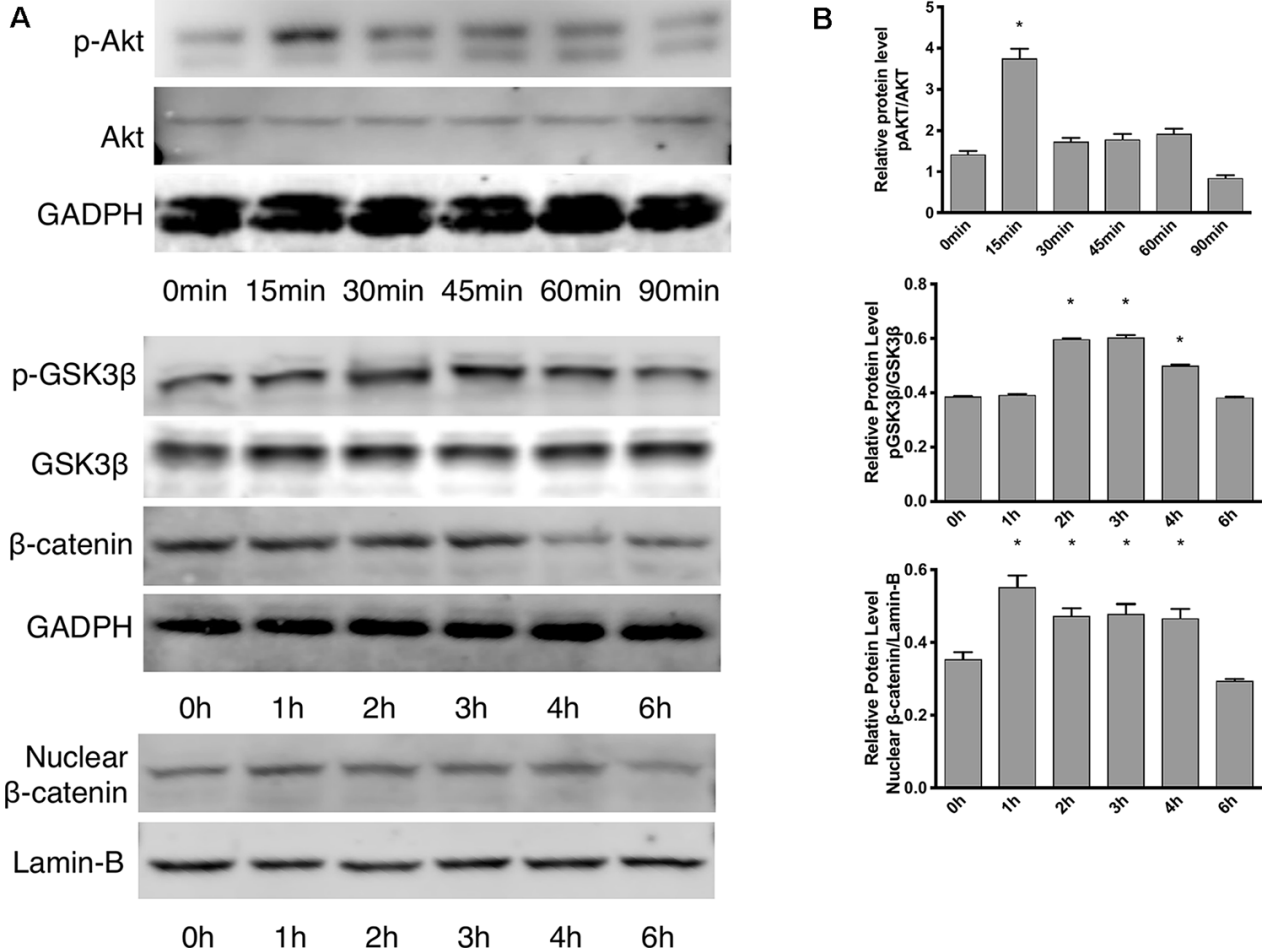
Effects of leptin on the Akt and Wnt signaling pathways. The concentration of leptin was 5 ng/ml. **(A)** Western blot assay for the phosphorylation of Akt, GSK3β, nuclear translocation of β-catenin. **(B)** Relative protein levels in (Panel **A**) of p-Akt, p-GSK3β and nuclear β-catenin, normalized with those of Akt, GSK3β, Lamin-B1 respectively. *indicated significant differences between different time groups vs. control group. P < 0.05. The experiments were conducted in triplicate.

According to the proliferation study and gene expression and *in vitro* angiogenesis assays, 5 ng/ml leptin was the ideal concentration for the following experiments. As shown in **Figure 3A**, in a short time, the phosphorylation of Akt was stimulated within 15 min. Leptin stimulated phosphorylation of GSK-3β and the release of β-catenin into the nucleus within specific times: GSK-3β phosphorylated at 2 h, and nucleus translocation occurred within 4 h (**Figure 3B**). This outcome demonstrates that both the Wnt and AKT signaling pathways were activated by leptin. Besides, adding dkk-1 also illustrated the same consequence (**Figure S2**).

### Effects of siRNAs on Akt and Wnt Pathways With Leptin

**Figure 4:**
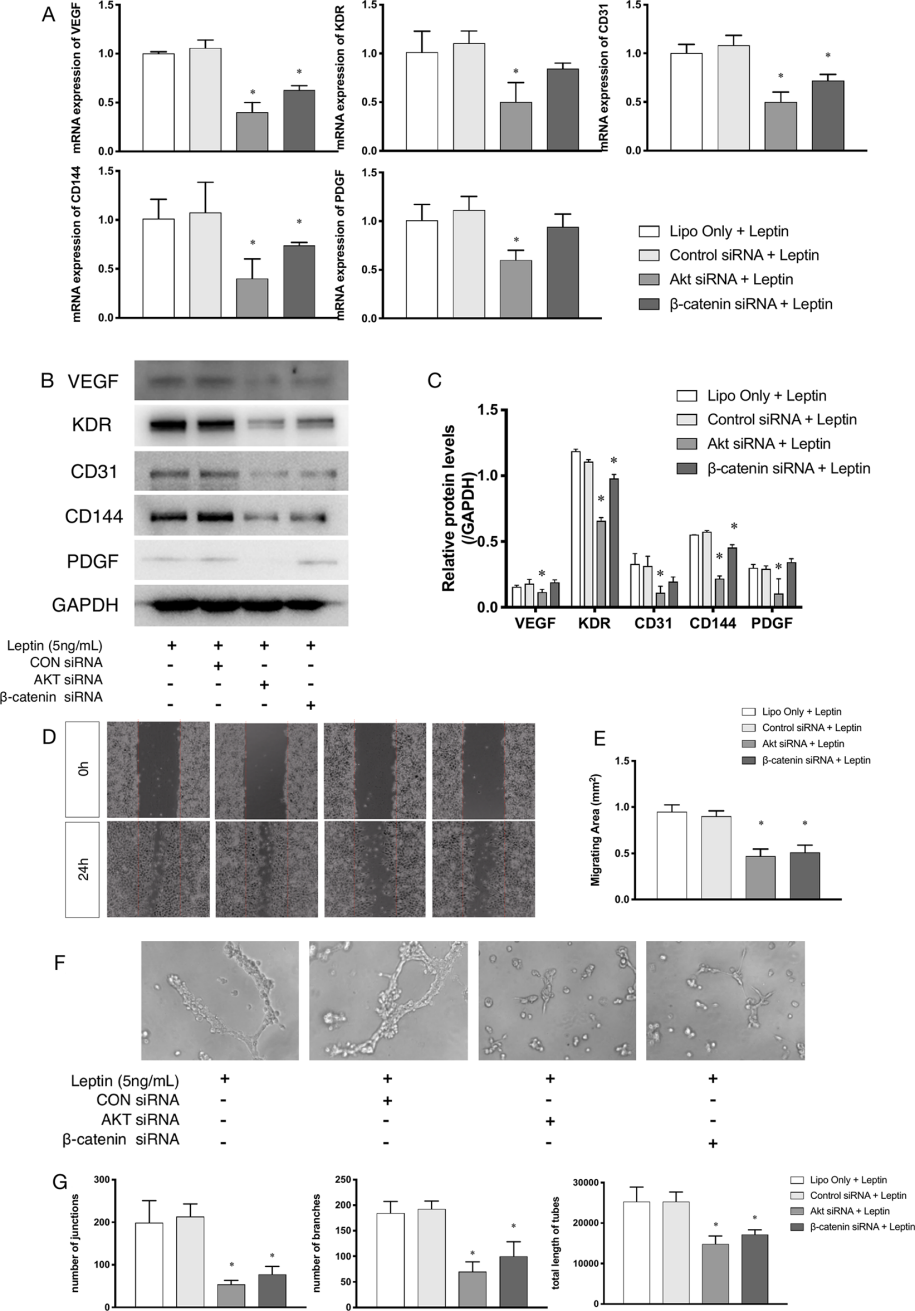
Angiogenesis gene expression, migration and tube formation with Akt siRNA or β-catenin siRNA. Cells were transfected with Akt siRNA (50 nM), β-catenin siRNA (50 nM) or control siRNA. Leptin (5 ng/ml) was added to each group. The mRNA and protein expression of VEGF, KDR, CD31, CD144, PDGF was investigated by quantitative real-time PCR, which Data were normalized to β-actin control **(A)** and Western blot assay **(B)**. **(C)** Relative protein levels of VEGF, KDR, CD31, CD144, PDGF, normalized with GAPDH of Panel *B*. **(D)** Cells were treated with different siRNA and were performed wound healing assay (original magnification, 40×). The red lines indicated wound edge at 0 h. **(E)** Migrating area (mm^2^) was calculated after 24h. **(F)** Cells were treated with different concentrations of leptin on BD Matrigel for 9h and capillary-like structures were examined (original magnification, 200×). **(G)** Number of junctions, number of branches and total length of tubes were valued. *indicated significant differences between siRNA-treated groups vs. control group. P < 0.05. The experiments were conducted in triplicate.

**Figure 5:**
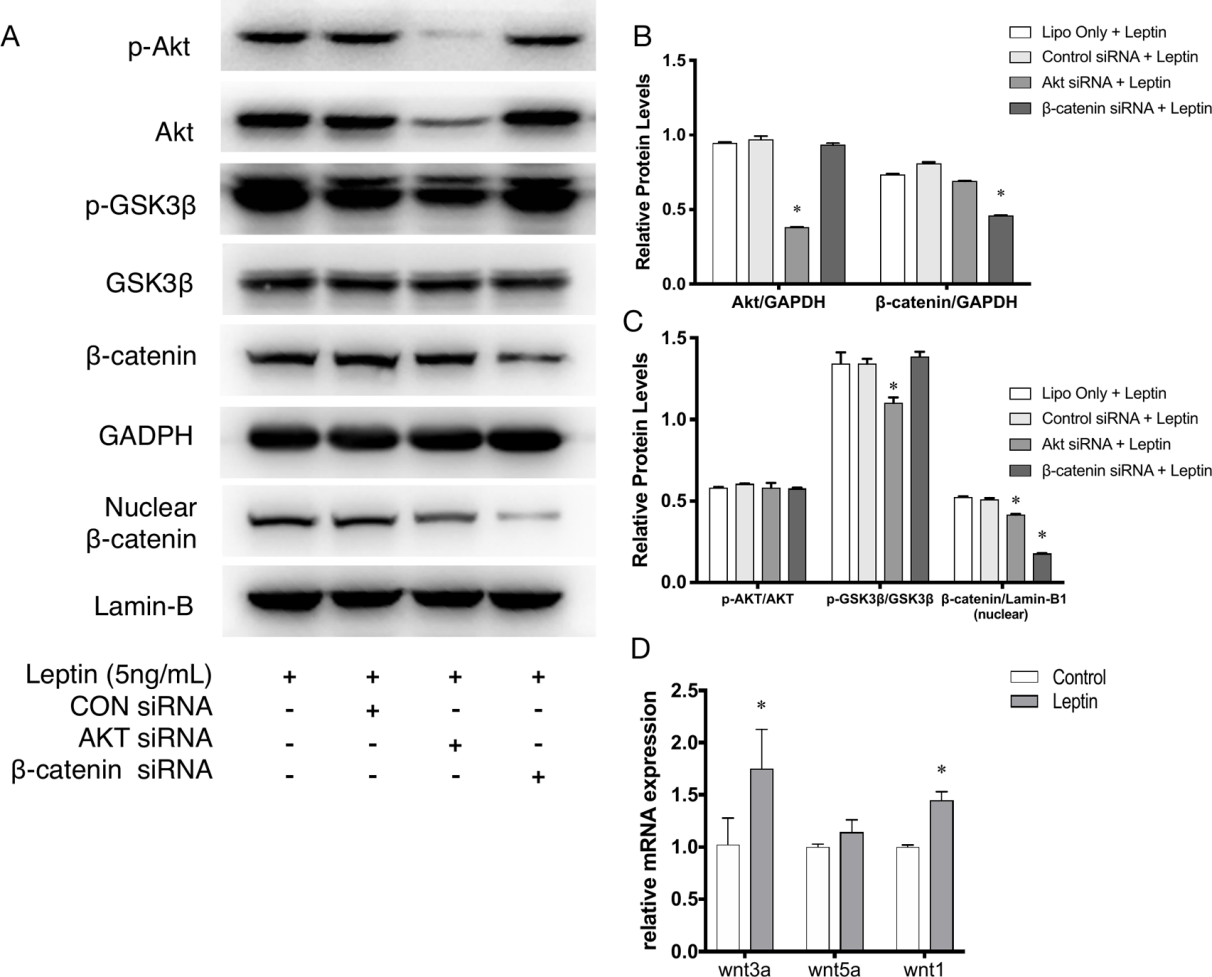
Phosphorylation of Akt, GSK3β, nuclear translocation of β-catenin applied with siRNAs. **(A)** Western blot assay for the phosphorylation of Akt, GSK3β, nuclear translocation of β-catenin. **(B)** Relative protein levels of total Akt and β-catenin in Panel *A*, normalized with GAPDH. **(C)** Relative protein level ratio changes of p-Akt, p-GSK3β and nuclear β-catenin in Panel *A*, normalized with those of Akt, GSK3β, Lamin-B1 respectively. *indicated significant differences between siRNA groups vs. control group. **(D)** Relative RNA expressions of Wnt1, Wnt3a and Wnt5a were evaluated by quantitative real-time PCR, which Data were normalized to β-actin control. *indicated significant differences leptin treated group vs. control group. P < 0.05. The experiments were conducted in triplicate.

siRNAs were used to confirm whether effects were induced by leptin *via* the Akt and Wnt signaling pathways. The knockdown efficiency was analyzed based on western blot (**Figures 5A, B**). With siRNAs of the Akt signaling pathway (Akt siRNA) and the Wnt signaling pathway (β-catenin siRNA), the expression of angiogenic genes, such as VEGF, KDR, CD31, CD144, PDGF, notably decreased (**Figures 4A–C**). Furthermore, the scratch healing (**Figures 4D, E**) and *in vitro* tube formation assays (**Figures 4F, G**) demonstrated that inhibition of the Akt and Wnt signaling pathways led to decreased angiogenesis, as was evidenced by the statistical analysis of the number of junctions, the number of branches, the total tube length and the percentage of wound healing size. The results suggested that the Akt and Wnt signaling pathways were involved in the stimulatory effects of leptin on angiogenesis in EA.hy926 endothelial cells.

### Effects of siRNA on Leptin-Induced Signaling Pathways in Endothelial Cells

Further, to demonstrate the influence of siRNAs on leptin-induced signaling pathways, Akt siRNA and β-catenin siRNA were added to endothelial cells to inhibit the Wnt signaling pathway and Akt pathway, respectively. Furthermore, Akt siRNA inhibited the phosphorylation of GSK-3β and the release of β-catenin into the nucleus, which suggested that activation of the Akt signaling pathway also played a crucial role in the canonical Wnt signaling pathway during leptin-induced activities (**Figures 5A, C**). To further investigate whether leptin induces Wnt expression, quantitative real-time PCR was performed. As was shown in **Figure 5D**, the expression of Wnt1 and Wnt3a were enhanced upon treatment with leptin. However, the expression of Wnt5a was not significantly improved. The results illustrated that leptin significantly induced the expression of Wnt1 and Wnt3a genes.

### Morphology of Electrospun Scaffolds

**Figure 6:**
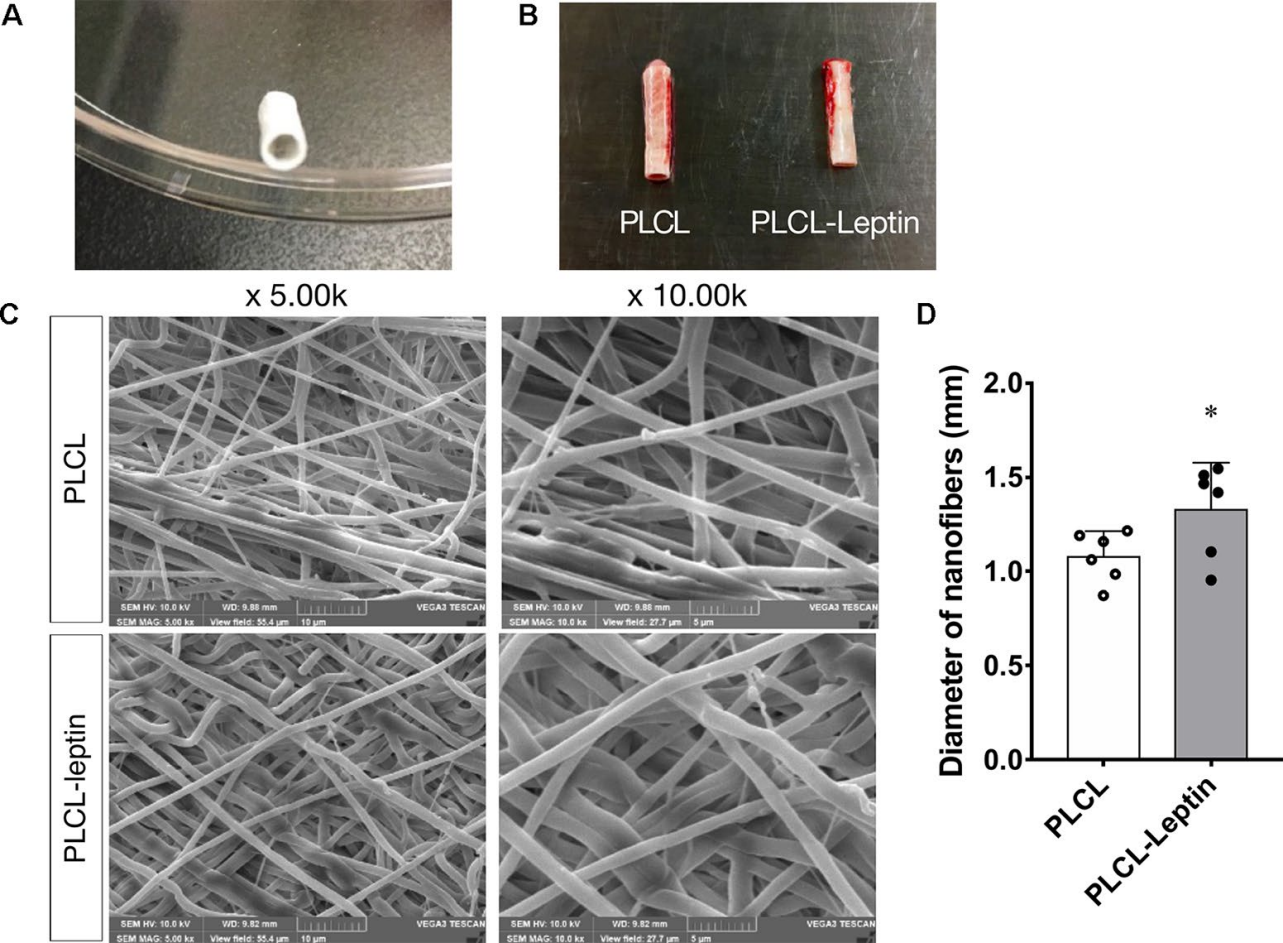
Characterization of PLCL scaffolds. **(A)** Leptin-immobilized PLCL scaffolds. **(B)** Explanted grafts. **(C)** SEM presentations of scaffolds. **(D)** Average diameter of scaffolds under SEM scanning. The experiments were conducted in triplicate. *Indicated significant differences between PLCL-leptin groups vs. PLCL control group. P < 0.05.

The Leptin-immobilized PLCL scaffold (**Figure 6A**) was implanted for 28 d and explanted (**Figure 6B**). The morphology of the fibers was characterized by SEM. Scanning electron microscopy (SEM) images of electrospun scaffolds were shown in **Figure 6C**, which shows PLCL and PLCL-leptin meshes with interconnected pores. The fiber size was uniform in PLCL/PLCL-leptin, the average diameter of PLCL fibers was 1.080 ± 0.254 mm, and the average diameter of PLCL-leptin fibers was 1.323 ± 0.411 mm (**Figure 6D**).

### HE Staining and Masson’s Trichrome Staining of Explanted Grafts

**Figure 7:**
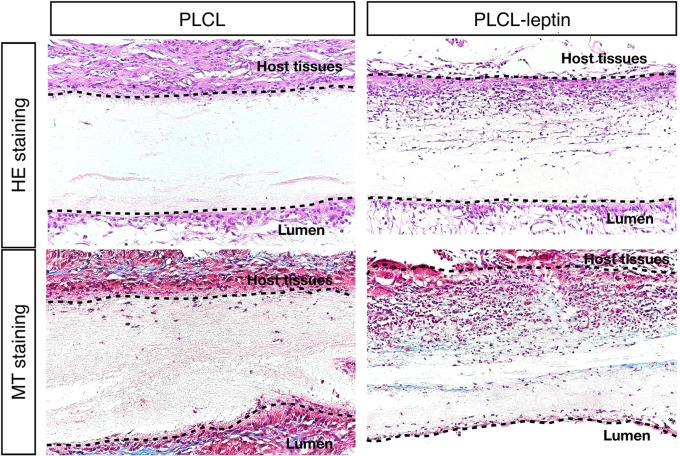
Evaluation of explanted graft in HE staining and Masson’s Trichrome (MT) staining. In MT staining of grafts, connective tissues were stained blue, nuclei dark red/purple, and cytoplasm red/pink. The experiments were conducted in triplicate.

After 28 days of implantation, scaffolds had adhered to surrounding tissues. Cell infiltration was much more significant in the PLCL-leptin group than that in the control group according to HE staining. Moreover, collagen was detected in the PLCL/PLCL-leptin group with MT staining. Subsequently, the density of the collagen deposits in the grafts was examined by trichrome staining, where collagen was stained blue (**Figure 7**). The results demonstrated that both groups of fibers maintained low density.

### Evaluation of Angiogenesis and Endothelial Cells in the Explanted Grafts

**Figure 8:**
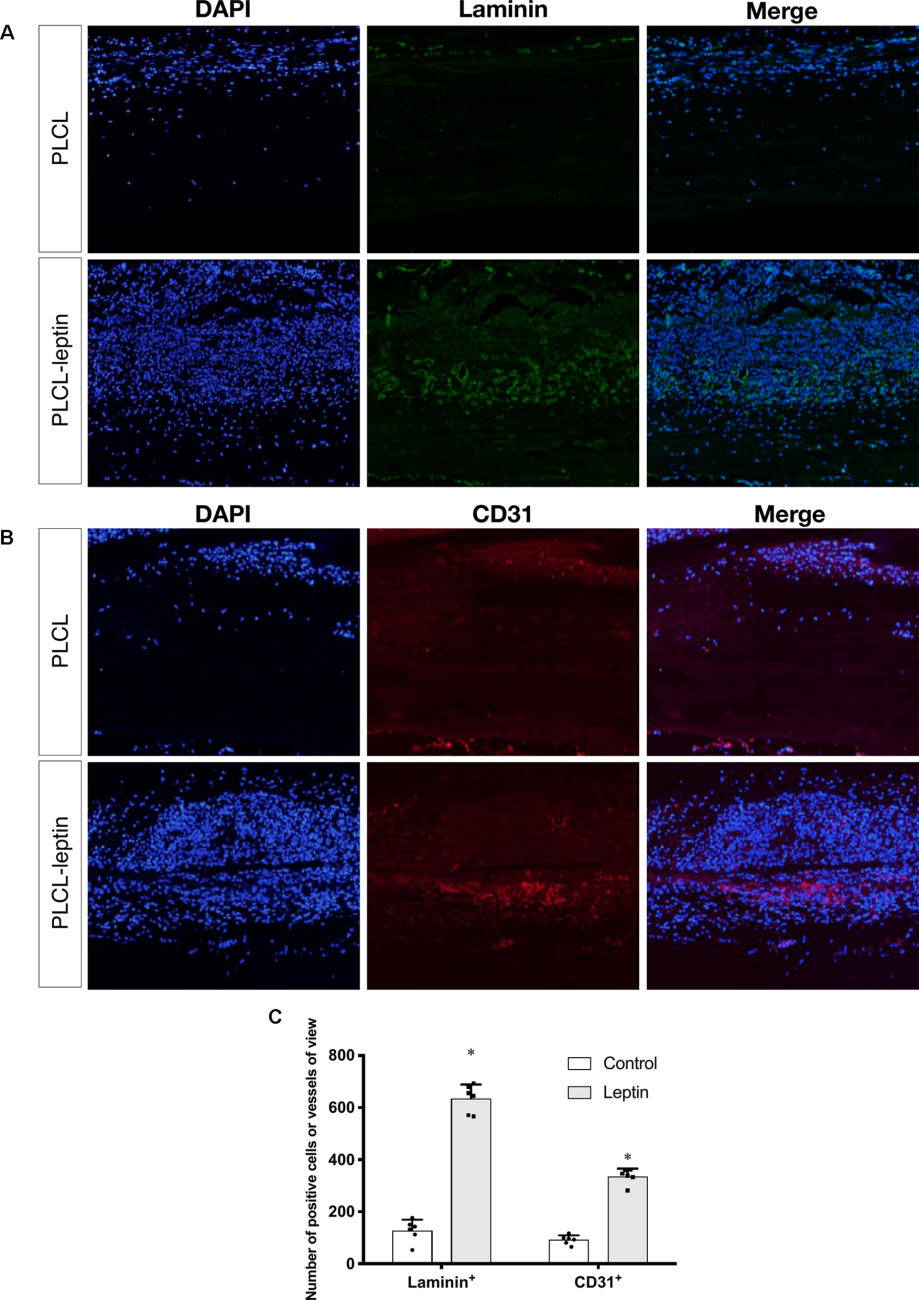
Evaluation of explanted graft in immunofluorescent staining. **(A)** Immunofluorescence staining of laminin^+^ vessels. **(B)** Immunofluorescence staining of CD31^+^ cells. **(C)** Counting of positive vessels or cells infiltrated in graft walls. Positive laminin^+^ and CD31^+^ cells were remarkably higher in PLCL-leptin group than control PLCL group. *indicated significant differences between PLCL-leptin groups vs. PLCL control group. P < 0.05. The experiments were conducted in triplicate.

In the immunofluorescence assay CD31 and laminin of grafts were stained to identify the infiltration of cells and evaluate angiogenic effects. The number of laminin^+^ ring structures and CD31^+^ cells infiltrated in graft walls were counted. It was apparent that the numbers of laminin+ vessels in PLCL-leptin grafts was significantly higher than in the control cells using microscopic and statistical analyses (**Figures 8A, C**). Moreover, the number of CD31+ cells in the PLCL-leptin grafts was significantly higher than in the control grafts (**Figures 8B, C**). Moreover, the numbers of both laminin+ vessels and CD31+ cells were higher at the exterior of the grafts than those at the interior.

## Discussion

Leptin, a hormone secreted by adipose cells, was first reported by (Zhang et al., 1994), and it has many functions in physiological processes. Leptin receptors on the arcuate nucleus of the hypothalamus regulate body energy homeostasis and appetite (Brennan and Mantzoros, 2006). Leptin can also induce angiogenesis. In 1998, leptin receptors were found on endothelial cells (Sierra-Honigmann, 1998). Apart from vascular cells, leptin promotes the cell proliferation of many other cell types, such as hemopoietic cells (Gainsford et al., 1996), dental mesenchymal stem cells (Um et al., 2011), skeletal myoblasts cells (Yu et al., 2014), murine embryonic stem cells (Kurtovic et al., 2015) and bone marrow stromal cells (BMSCs)(Zheng et al., 2017). Moreover, *in vitro* angiogenesis and *in vivo* chick chorioallantoic membrane assay were used to show that leptin promoted angiogenic processes (Bouloumie et al., 1998). The angiogenic property of leptin in the cornea of Zucker obese rat could be restored by transfection with the Ob-R gene (Jin et al., 2003). Some growth factors had also been studied in other studies. However, high cost, biological half-life, and uncertain side-effect limited their application in clinical use. For instance, VEGF has clearance half-life of less than 1 h following injection (Lee et al., 2003). Also they are easily to diffuse because of soluble property (Taraboletti and Giavazzi, 2004). Besides, in one study, VEGF could cause increased vascular permeability, led to severe edema in mice and caused high mortality rate (Epstein et al., 2001). In contrast, leptin costs less, and it has prolonged half-life because of innate ability (Hill et al., 1998). To these concerns, we chose leptin, a stable polypeptide for localized and sustained drug delivery.

In the present study, we performed fulfilled *in vitro* experiments to investigate the angiogenic effects of leptin. The CCK-8 proliferation assay indicated that cells proliferated in a dose-dependent manner. With 1 or 5 ng/ml of leptin, EA.hy926 endothelial cells grew much more rapidly. In addition, leptin also induced cells to transcribe mRNA related to angiogenesis. Expression of VEGF and KDR both increased, which suggested that the formation of blood vessels is stimulated. The expression of CD31, CD144, and PDGF, crucial players in cell migration and angiogenesis, were also enhanced. To detect migration and angiogenesis ability *in vitro*, we performed the scratch healing assay and the tube formation assay. Both assays indicated that 5 ng/ml was the most appropriate concentration of leptin for the angiogenesis ability of the EA.hy926 endothelial cells. Interestingly, endothelial cells exposed to a high concentration of leptin (i.e., 50 ng/ml) could had higher apoptosis rate than other concentrations.

However, there are few studies on how leptin promotes angiogenesis. (Vecchinoe et al., 2002) pointed out that leptin activated the Akt signaling pathway with nitric oxide as the mediator. Regarding cell migration, (Goetze et al., 2002) demonstrated that leptin activated the PI3K, Akt, and endothelial NO synthase and ERK pathways. Above all, the mechanism by which leptin promotes angiogenesis was still undefined. In recent years, the vital role of the Wnt signaling pathway in vascular stabilization and vascular disease has attracted much attention. The receptor of VEGF/PlGF, Fms-related tyrosine kinase 1 (Flt1), has been linked with cancer angiogenesis and found to mediate Wnt signaling pathway (Wang et al., 2018). Moreover, the enhanced expression of Wnt/β-catenin is accompanied by vascular reconstruction in the case of traumatic injury (Salehi et al., 2018). Furthermore, distinct mechanisms regulated Wnt-responsive GSK-3β and growth factor/Akt responsive GSK-3β, suggesting that GSK-3β has a crucial role in crosstalk between the Akt and Wnt signaling pathways (Valvezan and Klein, 2012), which must also be clarified in this study.

**Figure 9:**
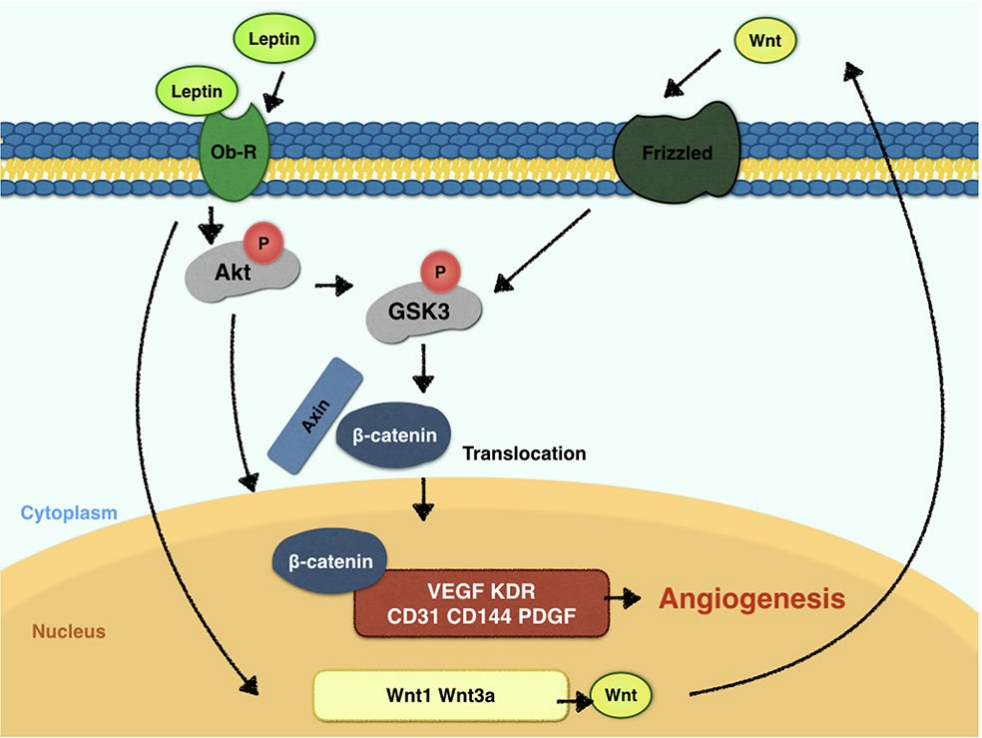
Signaling pathway model of leptin in EA.hy926 endothelial cells.

To elucidate the mechanism of leptin-induced angiogenesis, we investigated the cellular signaling pathways by which leptin affects EA.hy926 proliferation and differentiation. In the present study, the key mechanism is the activation of the Akt and Wnt/β-catenin pathways. We evaluated the phosphorylation of Akt, GSK-3β, and β-catenin in endothelial cell cultures after leptin treatment to examine the activation of the pathways. We provided evidence that leptin increased the phosphorylation of Akt and GSK-3β. Meanwhile, the stabilization and nuclear translocation of β-catenin were also magnified upon stimulation of leptin. β-catenin, which subsequently improved the expression of the target genes, such as VEGF, CD144, matrix metalloproteinase (MMP) 2, and MMP9, resulted in increased angiogenesis (Yan et al., 2012; Fernández et al., 2014). When knockdown of specific elements of the Akt and Wnt pathways by siRNAs and adding leptin, the expression of genes related to endothelial angiogenesis was inhibited. So was the cell migration and tube formation after treatment of siRNAs. These findings supported the idea that leptin-mediated angiogenesis is highly associated with the Akt and Wnt pathways. When the cells were transfected with Akt siRNA, the phosphorylation of GSK-3β and the translocation of β-catenin were significantly down regulated. Therefore, we speculated that activation of Akt pathway also activated the phosphorylation of GSK-3β and translocation of β-catenin. In addition, to determine whether leptin induces the expression of Wnt, we performed quantitative real-time PCR. It revealed that leptin also promoted the expression of Wnt1, Wnt3a and Wnt5a. The result agreed with the findings of previous research. Some studies reported a relationship between metastasis-associated protein 1 (MTA1) and the regulation of Wnt1 in endothelial and cancer cells (Kumar et al., 2010), and MTA1 might be a novel target for leptin (Yan et al., 2012). Another study also demonstrated that Wnt3a and insulin pathways both exhibited divergent and overlapping signaling activities in neuronal cells (Hooper et al., 2011). Moreover, Wnt5a was intimately related to angiogenesis through the noncanonical Wnt pathway (Shi et al., 2017), though no significant differences were presented in our study. Consequently, we may conclude that leptin activates the AKT/GSK-3β/β-catenin pathway and promotes the expression of Wnt1 and Wnt3a to activate canonical Wnt pathways, as was shown in **Figure 9**.

Over all, leptin has an angiogenic effect that is crucial for tissue regeneration. *In vivo*, we purposed to develop tissue engineering approaches to repair blood vessels. Engineered vascular grafts as arterial substitutes is proposed to overcome the limitations of autografts and other synthetic materials. Among cell-free vascular graft materials, biodegradable vascular grafts rely on host cells to regenerate blood vessels *in vivo*, without the need for pre-seeded cells. For vascular reconstruction, tissue-engineered vascular materials such as Darcon polyester grafts and polytetrafluoroethylene (PTFE) have been developed. However, thrombosis present as a great challenge for these transplant materials (Mehta et al., 2011). Various anticoagulants were applied to the materials, but with no significant improvement. Therefore, the solution goes back to the material itself. The materials of vascular grafts must be biocompatible with high elasticity, and PLCL, which is fabricated by electrospinning, has become a potential candidate. Because of its excellent elasticity and degradation ability, it has been used as a bioengineered graft in vascular tissue engineering (Mun et al., 2012; Mun et al., 2013). The electrospinning technique ensures great biocompatibility and offers unique opportunities for the efficient and reliable production of synthetic materials. Besides, the PLCL material is more robust because of its structure and modified hydrophobicity. Furthermore, PLCL also can also be used to regenerate other soft tissues, such as ligaments, skin, and cardiovascular soft tissue.

Therefore, in this study we chose a biodegradable material, PLCL. Because of its biodegradable and elastic properties, PLCL is a promising material for reconstructing blood vessels. Using the electrospinning technique, PLCL materials are also robust and can be efficiently produced. However, using the PLCL material alone as a tissue engineering material has certain limitations, one of which is the thickness. When the thickness of the material exceeds 150–200 μm, the diffusion of oxygen and nutrients is limited, which is detrimental to cell proliferation and migration (Jundziłł et al., 2017). The implanted material needs sufficient cellular infiltration and blood vessel formation to form a structurally stable, well-functioning network of neovascularization. In addition, to achieve successful tissue regeneration, the host stem cells need to reach an appropriate number, which means *in vivo* tissue regeneration might be hampered by limited infiltrate populations of host stem cells (Heissig et al., 2002; Lee et al., 2008). To solve this problem, we applied electrospinning technology to construct leptin-immobilized PLCL grafts, which could release leptin over a prolonged period, thus enhancing cell infiltration and angiogenic effects. In our study, conjugated leptin could be released *in vivo*, triggering a promising micro environment for cells and blood vessels. In addition to various chemotactic agents, leptin has been suggested as a novel potential bioactive molecule able to accelerate angiogenic functions of endothelial cells. In previous studies, leptin was encapsulated in hydrogels (Tadokoro et al., 2015) or a polyvinyl alcohol sponge (Anagnostoulis et al., 2008). These approaches were only used for small-scale processing and may not have the capacity for sustainable release. Therefore, we combined leptin and PLCL in bulk, achieving a copolymer that can deliver leptin over a prolonged period. According to (Jeong et al., 2004), PLCL was hydrolytically and enzymatically degraded during implantation. This indicated that PLCL was appropriate for extended *in vivo* implantation and as a suitable scaffold for leptin.

Under SEM, fibers were homogenous and seamless. The average diameter of PLCL-leptin fibers was larger than that of the PLCL control fibers, since that sheath layer increased the diameter of the scaffolds. Cell infiltration was the first maneuver in the regeneration of grafts through the vascularization processes (Zilla et al., 2007), and it was closely related to the degradation of grafts (Wu et al., 2012). Degradation was beneficial for cell infiltration. Host cells can infiltrate grafts and fulfill vascular generation. We demonstrated that cells infiltrated PLCL-leptin grafts much more than the PLCL control grafts. The sustainable release of leptin may provide a microenvironment suitable for cell proliferation and migration. Some cell types, such as vascular cells, stem cells, fibroblasts and myofibroblasts, might infiltrate into scaffolds. We also used MT staining to visualize collagen secreted by cells in scaffolds. Despite the limited density of collagen in the control and leptin groups 28 days after implantation, slightly more collagen was observed in the leptin groups than in the controls, which suggested that an extracellular matrix (ECM) was minimally produced in scaffolds with leptin. This phenomenon might imply that leptin has a lesser effect on secretion of collagen. Further identification was required to evaluate the activity of fibroblasts and myoblasts, and transmission electron microscope analysis of the grafts. Scaffold vascularization was assessed by immunofluorescence staining of laminin+ vessels and CD31+ cells. Cells that infiltrated scaffolds need oxygen and nutrition, and from this perspective, adequate vascularization is crucial. According to our findings, PLCL-leptin developed more laminin+ vessels and CD31+ cells than the PLCL control. The beneficial results were attributed extended release of leptin from scaffolds.

## Conclusion

This study shows that leptin promotes proliferation and differentiation of endothelial cells in a dose-dependent manner, with the effect culminating at a concentration of 5 ng/ml. During angiogenesis, the Akt and Wnt signaling pathways were also activated. In addition, the present study demonstrated that the conjunction of PLCL and leptin induced tissue regeneration *in vivo*, providing an encouraging solution for the sustained release of leptin in electrospinning meshes, which may be of great interest for tissue engineering applications.

## Data Availability Statement

The datasets generated for this study are available on request to the corresponding author.

## Ethics Statement

The animal study was reviewed and approved by The Ethics Committee of Ninth People’s Hospital Affiliated to Shanghai Jiao Tong University School of Medicine.

## Author Contributions

LX, BF, and XM conceived and designed the experiments. FY, LL, and RF performed experiments, analyzed the results, and made the figures and tables. XW, TW, WS, and ZG contributed to the scientific discussions. FY and LX wrote the paper.

## Conflict of Interest

The authors declare that the research was conducted in the absence of any commercial or financial relationships that could be construed as a potential conflict of interest.

## Supplementary Material

Figure S1 The apoptosis measurement. Cells were detected by flow cytometry after treating with different concentration of leptin for 24 h. *indicated significant differences between leptin-treated groups vs. control group. P < 0.05. The experiments were conducted in triplicate.

Figure S2 Angiogenesis gene expression, migration, tube formation and protein expression with Dkk-1. **(A)** Evaluation of the leptin-induced proliferation of EA.hy926 endothelial cells at 24h. The mRNA and protein expression of VEGF, KDR, CD31, CD144, PDGF was investigated by quantitative real-time PCR, which data were normalized to β-actin control. **(B)** Cells were treated with leptin or leptin + Dkk-1 on BD Matrigel for 9h and capillary-like structures were examined (original magnification, 200×). **(C)** Number of junctions, number of branches and total length of tubes were valued. **(D)** Cells were treated with leptin or leptin+Dkk-1 and were performed wound healing assay (original magnification, 40×). **(E)** Migrating area (mm^2^) was calculated after 24h. **(F)** Phosphorylation of Akt, GSK3β, nuclear translocation of β-catenin applied with leptin or leptin + Dkk-1. **(F)** Relative protein levels of total Akt and β-catenin in Panel **A**, normalized with GAPDH, Relative gray value ratio changes of p-Akt, p-GSK3β and nuclear β-catenin in Panel A, normalized with those of Akt, GSK3β, Lamin-B1 respectively. *indicated significant differences between leptin group vs. leptin+Dkk-1 group. P < 0.05. The experiments were conducted in triplicate.
