# Supplementary figures and images for: Leptin-Induced Angiogenesis of EA.Hy926 Endothelial Cells via the Akt and Wnt Signaling Pathways In Vitro and In Vivo
Source: Front Pharmacol. 2019 Oct 31;10:1275. doi: 10.3389/fphar.2019.01275 (PMC6836761; doi:10.3389/fphar.2019.01275)

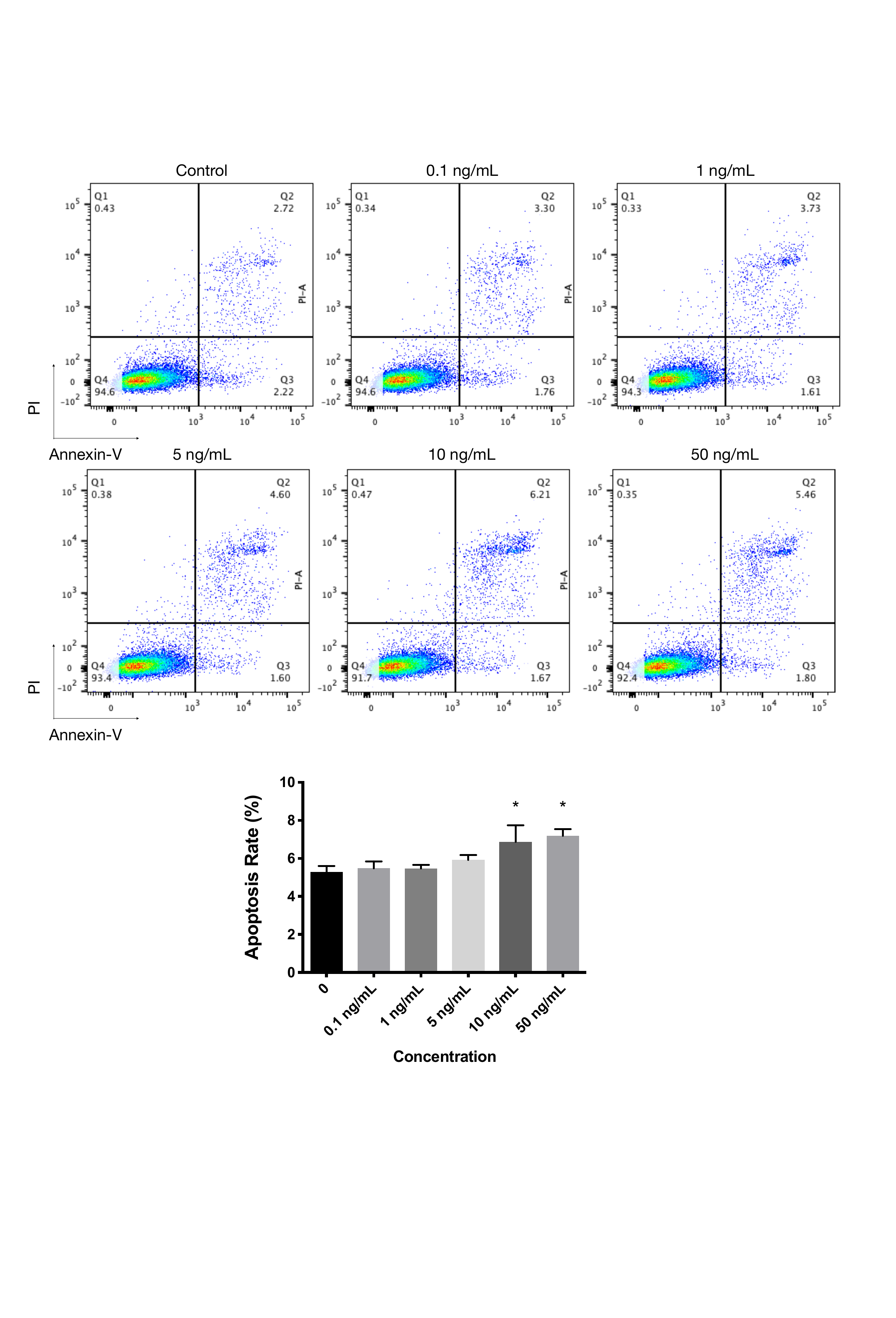

Supplement: Figure S1 — The apoptosis measurement. Cells were detected by flow cytometry after treating with different concentration of leptin for 24 h. *indicated significant differences between leptin-treated groups vs. control group. P < 0.05. The experiments were conducted in triplicate. [file Image_1.tiff]

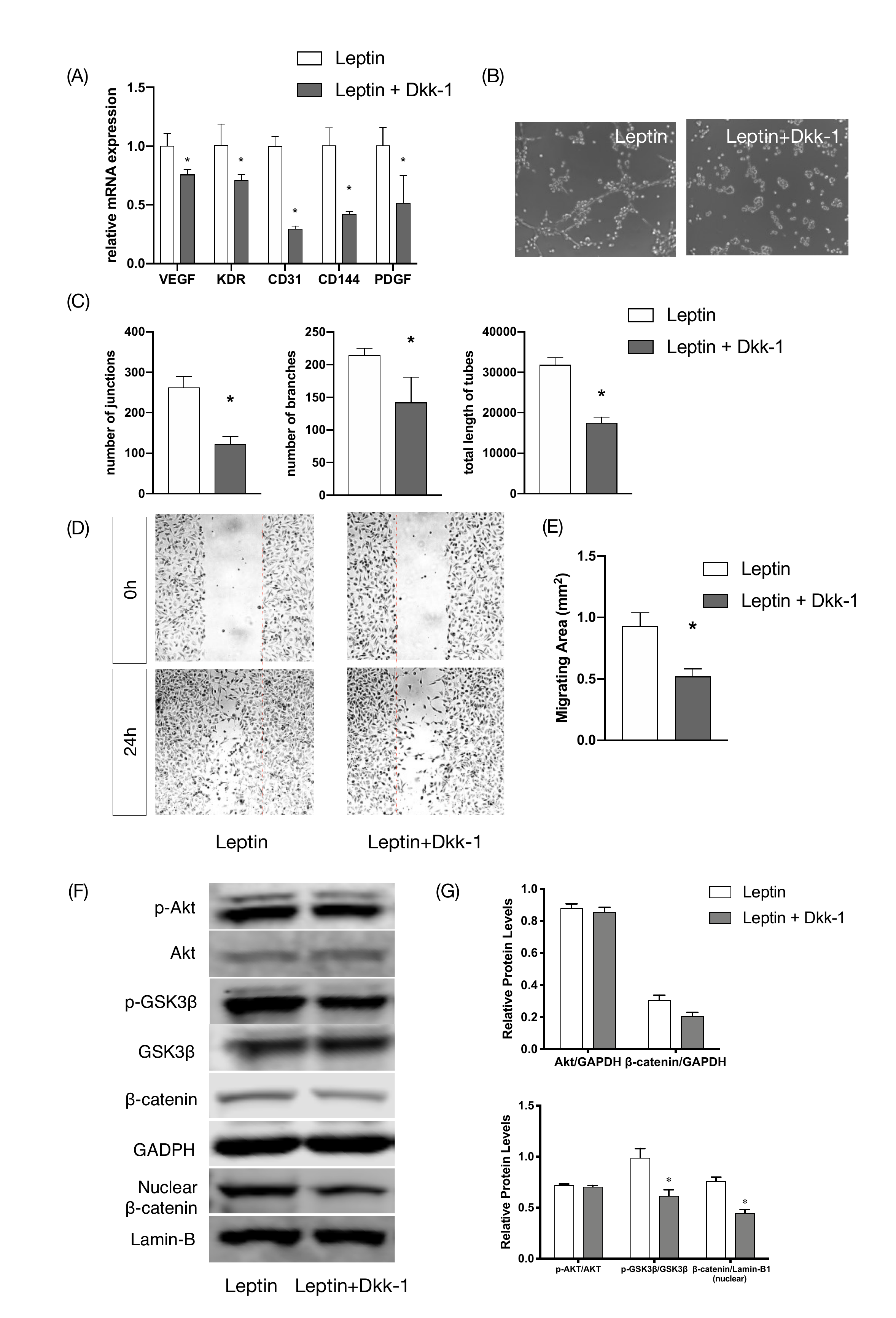

Supplement: Figure S2 — Angiogenesis gene expression, migration, tube formation and protein expression with Dkk-1. (A) Evaluation of the leptin-induced proliferation of EA.hy926 endothelial cells at 24h. The mRNA and protein expression of VEGF, KDR, CD31, CD144, PDGF was investigated by quantitative real-time PCR, which data were normalized to β-actin control. (B) Cells were treated with leptin or leptin + Dkk-1 on BD Matrigel for 9h and capillary-like structures were examined (original magnification, 200×). (C) Number of junctions, number of branches and total length of tubes were valued. (D) Cells were treated with leptin or leptin+Dkk-1 and were performed wound healing assay (original magnification, 40×). (E) Migrating area (mm2) was calculated after 24h. (F) Phosphorylation of Akt, GSK3β, nuclear translocation of β-catenin applied with leptin or leptin + Dkk-1. (F) Relative protein levels of total Akt and β-catenin in Panel A, normalized with GAPDH, Relative gray value ratio changes of p-Akt, p-GSK3β and nuclear β-catenin in Panel A, normalized with those of Akt, GSK3β, Lamin-B1 respectively. *indicated significant differences between leptin group vs. leptin+Dkk-1 group. P < 0.05. The experiments were conducted in triplicate. [file Image_2.tiff]
